# Urban livestock-keeping and dengue in urban and peri-urban Hanoi, Vietnam

**DOI:** 10.1371/journal.pntd.0007774

**Published:** 2019-11-26

**Authors:** Frida Jakobsen, Thang Nguyen-Tien, Long Pham- Thanh, Vuong Nghia Bui, Hung Nguyen-Viet, Son Tran- Hai, Åke Lundkvist, Anh Bui- Ngoc, Johanna F. Lindahl

**Affiliations:** 1 Uppsala University, Department of Medical Biochemistry and Microbiology, Uppsala, Sweden; 2 International Livestock Research Institute, Hanoi, Vietnam; 3 National Institute of Veterinary Research, Hanoi, Vietnam; 4 Hanoi University of Public Health, Hanoi, Vietnam; 5 National Institute of Hygiene and Epidemiology, Hanoi, Vietnam; 6 Swedish University of Agricultural Sciences, Department of Clinical Sciences, Uppsala, Sweden; USDA-ARS Center for Medical Agricultural and Veterinary Entomology, UNITED STATES

## Abstract

Urban livestock provides an important source of food and income, but it may increase the risks for disease transmission. Vectors, such as mosquitoes, might increase and thereby cause an enhanced transmission of infectious diseases, such as dengue fever; considered the most important mosquito-borne viral disease globally. This cross-sectional study evaluated the awareness of dengue fever and investigated how the presence of dengue vectors is affected by the keeping of livestock in urban households in the city of Hanoi, Vietnam.

From February to March 2018, during the season of lowest occurrence of dengue in Hanoi, 140 households were interviewed, of which 69 kept livestock. A general trend was observed; respondents living in the Dan Phuong district, a peri-urban district, had better knowledge and practice regarding dengue as compared to the urban Ha Dong district. In total, 3899 mosquitoes were collected and identified, of which 52 (1.33%) were *Aedes* species. A significant difference between the two districts was observed, with more households in Ha Dong having *Aedes* spp. mosquitoes (p = 0.02) and a higher incidence of dengue fever (p = 0.001). There was no significant association between livestock-rearing and the presence of *Aedes* spp. mosquitoes (p = 0.955), or between livestock-rearing and the incidence of dengue fever (p = 0.08).

In conclusion, this study could not find any indication that households keeping livestock were at higher risk of dengue virus infections in Hanoi during the season of lowest occurrence of dengue, but clearly indicated the need of more information provided to urban inhabitants, particularly on personal protection.

## Author summary

In many rapidly growing tropical cities, urban livestock keeping are an important provider of food for the urban population, as well as contributing to livelihoods. The high density of both humans and animals in cities can however contribute to diseases being transferred from animals to humans. Less studies have so far been performed on how the urban livestock keeping can contribute to increased number of vectors for diseases that are transmitted between humans in cities, such as dengue fever. In this study we explored the urban livestock keeping in Hanoi, the capital of Vietnam, and the mosquitoes that transfer the dengue virus. We found that more of these mosquitoes were present in the more central homesteads than in the peri-urban areas, and that livestock keeping did not increase the number. The central households had experienced more dengue fever cases, and we also found that the peri-urban households were much more aware about how to protect themselves from the disease. This indicated a need to increase the information campaigns primarily in more central parts of the cities.

## Introduction

Urbanization and livestock intensification are closely connected. Livestock provides an important source of food for the city, as well as income for the farmers. With more humans and animals living in close vicinity, vectors such as mosquitoes may increase in numbers, and thus cause enhanced transmission of infectious diseases, e.g. dengue fever, Japanese encephalitis (JE) and malaria [1,2]. Dengue fever was ranked the most important mosquito-borne viral disease in the world in 2012, and outbreaks of the disease repeatedly occur throughout most tropical countries [3]. Through an evidence based-scoring system and modelling, 3.97 billion people are estimated at risk of being infected with dengue virus in 128 countries around the world [4]. Over the past 50 years the incidence of dengue fever has increased 30-fold, and the global burden is today predicted to be 390 million (284–528, 95% CI) dengue virus infections each year. Of these, 96 million (67–136) are apparent infections (i.e. clinical disease) of different levels of severity. In 2010, Asia suffered around 70% of the total number of dengue virus infections [3,5].

Dengue fever was initially reported in Vietnam in 1959 and has since then become endemic across the country [6]. In 2010, Vietnam had among the highest cumulative incidence of dengue fever (14,469 incidence/100,000 population) in the Western Pacific region [6]. Several outbreaks of dengue fever have been reported in Vietnam over the years and major outbreaks have occurred approximately every ten years (1987, 1998, 2009, 2017). The 2009 outbreak in Hanoi reported 16,263 dengue cases, with 121 cases per 100,000 people, which was 6.7-times higher as compared to the same period in 2008. The latest dengue fever outbreak in Hanoi occurred in 2017 with 37,651 cases and 7 deaths reported [7,8].

Vietnam is a tropical country in the Western Pacific region. In 2016, 34% of the total population were living in urban settings, a number that increases each year [9]. The capital, Hanoi, is the second most populated city with an average population of 7.3 million people [10]. There is also a high livestock population in Hanoi with 22.3 thousand buffaloes, 129.5 thousand cattle (beef and dairy), 1.6 million pigs and 24.4 million poultry according to data from General Statistics Office of Viet Nam [10].

Several earlier studies have reported weather and climate components as important risk factors for the incidence of dengue fever and vector survival in Vietnam [11–14]. Other findings in Vietnam suggest that keeping an animal shelter, a garden and garbage close to the households were similarly associated with a higher incidence of dengue fever. Also, farmers were shown to have an almost eight times higher risk of dengue virus infection (relative risk 7.94; 95% CI 2.29–27.55) [15]. It has also been suggested that there is an association between the presence of dengue virus-specific IgG antibodies and the management of pigs. The reason for this association may be the formation of habitats for *Aedes* larvae when raising pigs, although that was not studied in detail. However, there is a huge gap in knowledge in this area and thereby an urgent need for further investigations [16].

Livestock keeping can influence the number of vectors close to humans [17], and thus it is hypothesized that a high number of livestock and humans in close contact may increase the risk of dengue virus transmission, particularly if the keeping of livestock is associated with the presence of larval habitats and higher numbers of mosquitoes. The keeping of livestock may create more breeding sites for mosquitoes due to incomplete sanitation, increased water usage, and provide ample opportunities for blood feeding [17]. The dengue virus vectors (*Aedes aegypti* and *Ae*. *albopictus*) are able to breed in diverse breeding sites, including naturally formed water bodies, such as tree holes, but also any kind of man-made container [18]. These breeding sites could be expected to increase around animals. It is therefore of great importance to understand how the presence of dengue virus vectors are affected by the keeping of livestock in urban areas since previous studies are scarce. The aims of this study were therefore to investigate the presence of dengue virus vectors (*Aedes aegypti* and *Ae*. *albopictus*) during low transmission season, and how this is affected by the keeping of livestock in urban environments in Hanoi, Vietnam, as well as gaining a better understanding of the public awareness of dengue fever.

## Methods

**Fig 1:**
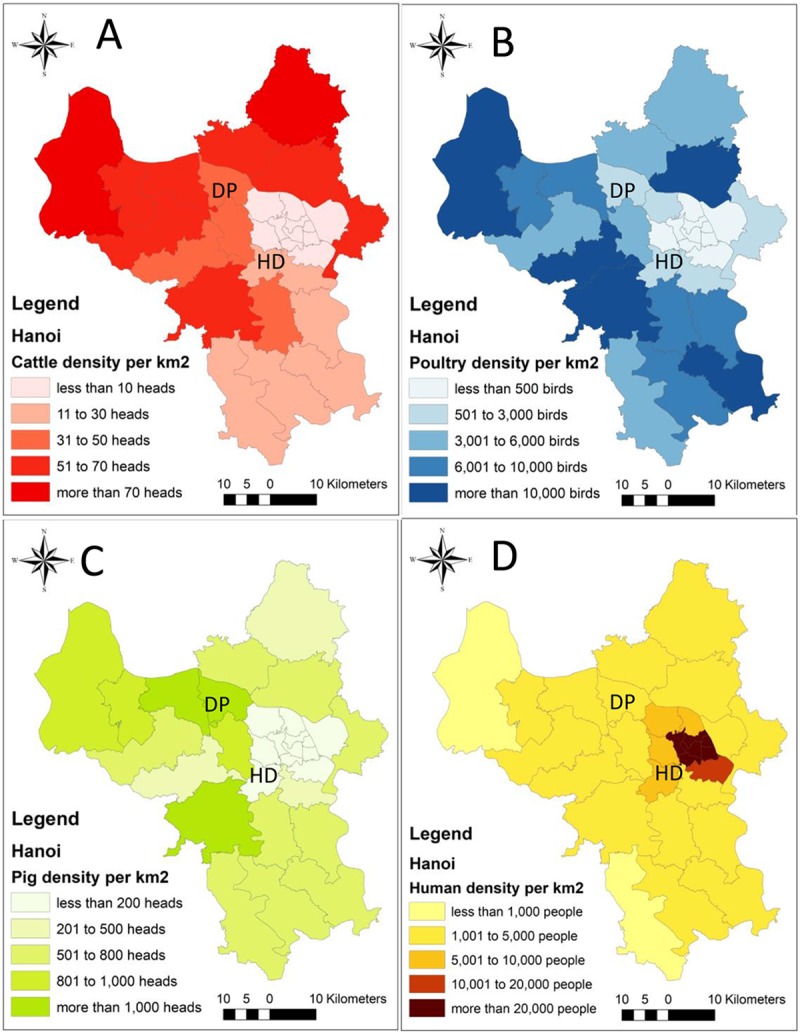
The population density of livestock (cattle and poultry) in the districts of Hanoi city (population/km2). The data was obtained from 2017 from the Hanoi sub-Department of Animal Health. A. Cattle population density B. Poultry population density C. Pig population density D. Human density. Maps created using ArcGIS version 10.3 ArcMap (ESRI, Redlands, CA). HD: Ha Dong. DP: Dan Phuong.

**Fig 2:**
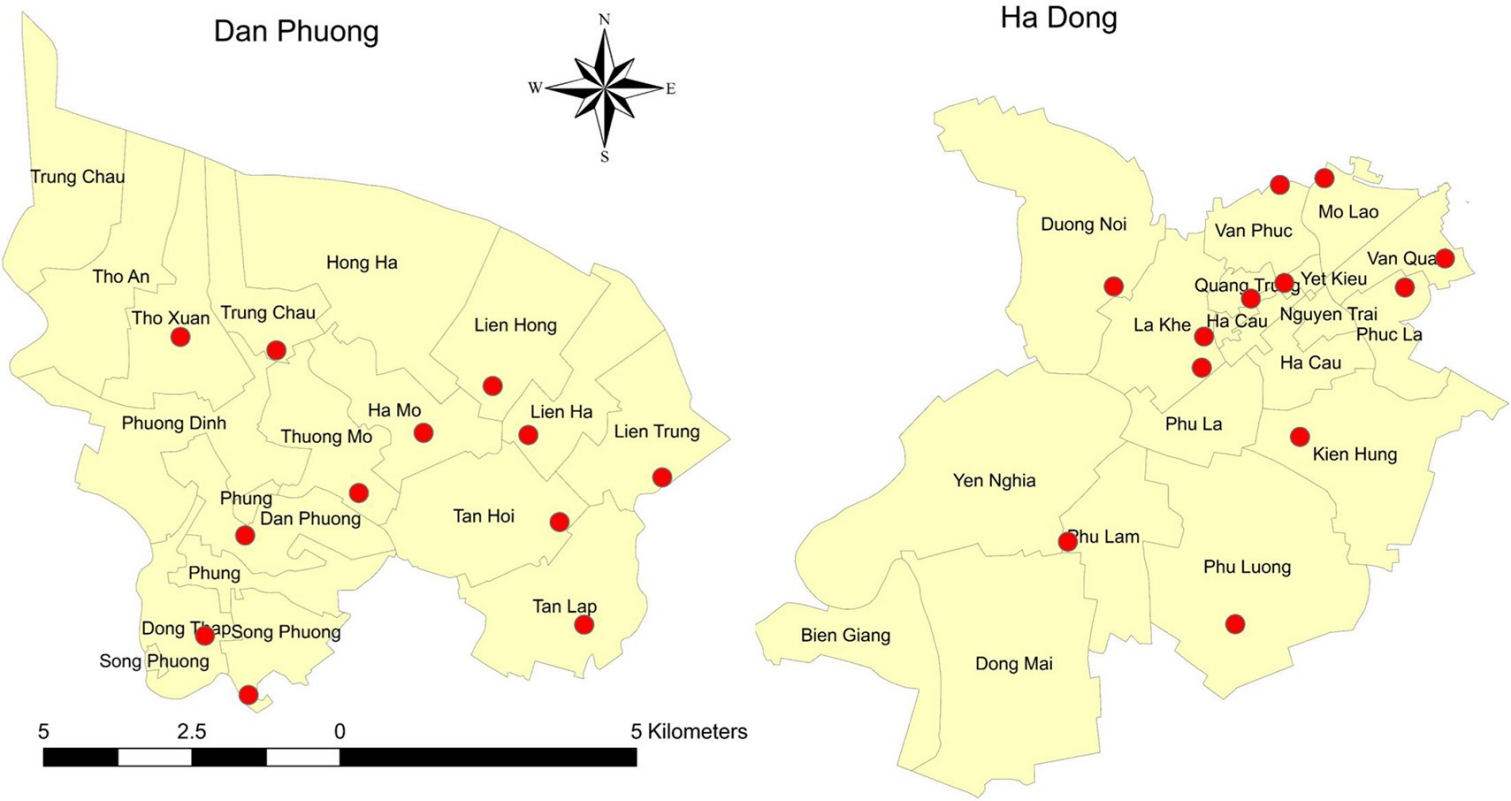
Maps showing the 12 randomly selected GPS-points for the two selected districts; Dan Phuong and Ha Dong. Maps created using ArcGIS version 10.3 ArcMap (ESRI, Redlands, CA).

A cross-sectional study was implemented in two districts of Hanoi, Vietnam, from February to March 2018 (Strobe checklist in Supplemental material S1 Text), which is the season of lowest occurrence of dengue in Hanoi [19]. The two districts, Ha Dong and Dan Phuong, were selected based on livestock population data from the Hanoi sub-department of animal health in 2017. Ha Dong is located in the central part of Hanoi with a low livestock population density and a high human population density (6037.3 persons/km^2^), while Dan Phuong is a peri-urban area with a higher livestock population density (Figs 1 and 2) and a lower population density (1996.1 persons/km^2^) [10].

### Sample size and selection of households

To calculate the required sample size, 10% of the households were presumed to have dengue vectors, which was based on our previous results [17]. With an anticipated precision of 5 and a 95% confidence level, the sample size was calculated to 139 households. This sample size would also be enough to detect a difference of 20% with a power of 80% between the non-livestock- and the livestock-keeping households. In each of the two districts, 70 households were interviewed, and the same number of livestock-keeping and non-livestock-keeping households were identified through selection of random GPS points and from the help of local veterinarians. In this study, a household with livestock was defined as having at least one larger livestock species e.g. cattle or pig, or at least 10 smaller animals e.g. chicken or duck. 16 random GPS points were identified in each district using ArcGIS 10.4 and the distance between each point was set to 1.5 km. Out of these 16 random GPS points, 12 were randomly selected in each district to generate 72 households per district (Fig 2). Three households without and three households with livestock were identified for each GPS point. In the Ha Dong district, the 12 selected points were located in central areas where there were no livestock was present, hence, all 12 selected points were not used and instead, local veterinarians identified communes and households with livestock. Therefore, only 7 GPS points (7–12 households per point) were included to generate the total number of 70 households. In the Dan Phuong district, close to the selected GPS points, the veterinarians randomly selected households with and without livestock for participation in the study. For both districts, if the selected household was not willing to participate, the interviewer continued visiting neighbouring households until a consenting household was found.

### Questionnaire and interviews

The questionnaire was first developed in English and later translated into Vietnamese. A pilot study was performed before conducting the complete study through interviewing three households located in the Long Bien district, Hanoi. The field work was conducted during March 2018 and in each household, a person above 18 years of age that lived permanently in the household was interviewed in Vietnamese after giving their informed consent. The data collected on animals kept in the households was in reference to the situation at the time of interviewing. The households were interviewed with a structured questionnaire (Supplement materials S2 Text and S3 Text) regarding details about the household, management of the livestock and their awareness of dengue.

### Mosquito and larvae collection and identification

The households were asked for their consent for the collection of mosquitoes using a backpack aspirator. The aspirator was used for 5 min ±30s inside and outside the household. Mosquitoes were collected close to the livestock in the livestock-keeping households, while in households without livestock, the outdoor collection was performed around the garden. It was not possible to collect mosquitoes in all households, and these were handled as missing data. Each household was searched for water-filled open containers, and larvae were collected if present. The larvae were collected using a larvae collection tool kit and through the following standard methods of the National Institute of Hygiene and Epidemiology (NIHE), Vietnam [20]; for deeper containers, a hand net was lowered in the water while circulating for 5 turns. Shallow containers were observed by eye and if larvae were observed, they were collected using a pipette.

The mosquitoes were counted and identified to genus with those belonging to Aedes further identified to two species, *Aedes aegypti* and *Ae*. *albopictus*. The larvae were identified to genus (*Anopheles*, *Culex* or *Aedes*) through light microscopy following standard operating procedures at the NIHE [21].

### Statistical methods and ethical considerations

The data is presented with descriptive statistics and univariable tests of association. The presence of *Aedes* vectors was handled as a binary variable. Missing data was not imputed. To decide if there was a difference between the two districts concerning the knowledge, attitude and practice questions, Chi^2^-test was performed. When analysing the association between different factors (eg. district, livestock keeping, *Aedes* spp. mosquitoes etc.) and confirmed dengue cases in 2017, Chi^2^-test was performed. When analysing the association between presence of *Aedes* spp. mosquitoes and the two districts, Chi^2^-test was also used. Multivariable logistic regression was done to evaluate the association between the risk of dengue in the household, with the independent variables: livestock keeping in the family; knowledge about dengue, as the sum of all symptoms and mosquito breeding grounds correctly identified; and district. All statistical analysis was performed using STATA 14 (StataCorp Ltd, College Station, Texas, US). Maps were created using ArcGIS version 10.3 ArcMap (ESRI, Redlands, CA). Data is provided in supplement materials S1 Data.

The study protocol was approved by the ethical committee of Hanoi University of Public Health (HUPH) (approval reference No.: 048/2018/YTCC-HD3). All participants were informed that the participation in the study was voluntary and that they had the possibility to drop out of the study at any time. A written informed consent was obtained from all participants before conducting the interview.

## Results

### Socio-demographic characteristics of the households

One hundred and forty households were interviewed. Among those, 66 (47.14%) of the respondents were males and 74 (52.86%) were females. The age of the respondents varied between 17 and 85 years, with most respondents at an age of 51–60 years. There was no significant difference of the education levels between the genders. Slightly more of the females had finished primary, secondary, high school and college/university, respectively as compared to the males. Regarding occupations of the respondents, the majority (69.29%) were farmers and only one respondent (0.71%) had some kind of medical background or medical education. Of the respondents, 27.94% had other occupations, which included working in: business, in a store, and as a teacher. Concerning the gender of the household heads, 72% of them were male and 28% were female. More than half of the households had children. The number of children varied between 1–5 and almost all households had at least one child under the age of 15 (Table 1).

**Table 1:** Socio-demographic characteristics of the households in the Dan Phuong and Ha Dong districts.

	*Households (%)* *Dan Phuong*	*Households (%)* *Ha Dong*	*Households (%)* *all*
***Respondent gender***			
Male	33 (46.48)	33 (47.83)	66 (47.14)
Female	38 (53.52)	36 (52.17)	74 (52.86)
***Respondent age***			
17–30	5 (7.04)	8 (11.59)	13 (9.29)
31–40	11 (15.49)	11 (15.49)	22 (15.71)
41–50	13 (18.31)	14 (20.29)	27 (19.29)
51–60	27 (38.03)	21 (30.43)	48 (34.29)
61–70	12 (16.90)	11 (15.94)	23 (16.43)
71–85	3 (4.23)	4 (5.80)	7 (5.0)
***Respondent education level***			
No education	5 (7.25)	6 (8.82)	11 (7.86)
Primary school	11 (15.94)	12 (17.65)	23 (16.43)
Secondary school	29 (42.03)	39 (57.35)	68 (48.57)
High school	20 (28.99)	8 (11.76)	28 (20.0)
College/University	4 (5.80)	1 (1.47)	5 (3.57)
***Respondent occupation***			
Farmer	48 (69.57)	49 (71.01)	97 (70.29)
Medical profession	0 (0)	2 (2.90)	2 (0.72)
Other	21 (30.43)	18 (26.09)	38 (27.14)
***Household head gender***			
Male	53 (74.65)	47 (68.12)	100 (71.43)
Female	18 (25.35)	22 (31.88)	40 (28.57)
***Children <15 in household***			
Yes	43 (61.43)	48 (70.59)	91 (65.94)
No	27 (38.57)	20 (29.41)	47 (34.06)
***Livestock keeping***			
Yes	36 (50.70)	33 (47.83)	69 (49.29)
No	35 (49.30)	36 (52.17)	71 (50.71)

### Livestock information

In total, 69 households with livestock were interviewed, of which 33 were located in Ha Dong and 36 in Dan Phuong. The households mainly managed pigs and/or chicken. People living in Dan Phuong district managed more pigs (2881 in total, average 120 pigs/household) compared to the 449 pigs in total and an average of 23.6 pigs/household in Ha Dong. Ha Dong district managed more chickens, 3464 in total, and an average of 157.5 chickens/household, as compared to 1086 chickens in total and an average of 40.2 chickens/household in Dan Phuong. Ducks, cattle and goats were uncommon in both districts, and no households kept buffalos (Table 2).

**Table 2:** Livestock-keeping households in Dan Phuong and Ha Dong districts.

	*Dan Phuong district*					*Ha Dong district*				
*Animals*	*Pig*	*Chicken*	*Duck*	*Cattle*	*Goat*	*Pig*	*Chicken*	*Duck*	*Cattle*	*Goat*
***Number of households***	24	27	3	2	1	19	22	2	2	0
***Mean number of animals***	120	40.2	170	1.5	20	23.6	157.5	3	4	0
***Management (% of households)***										
*Fenced in*, *outdoor*	100	96.3	100	100	100	100	95.45	50	100	0
*Free roaming*	0	3.7	0	0	0	0	4.55	50	0	0

The majority of all animals were kept fenced outdoors in both districts, but some of the households kept the animals free roaming. None of the households kept the animals indoor, or partly indoor (Table 2).

### Sources of communication for dengue fever

The majority (97.9%) of the respondents had earlier heard about dengue fever. Regarding the sources of information on dengue fever; TV (80.6%), loud speakers (55.0%) and health care workers (44.2%) were stated as the most important sources. Communication material (23.3%) and the internet (3.2%) were the least important sources for information on dengue fever. Between the two districts there was a significant difference for TV (p = 0.006), loudspeaker (p<0.001) and health care worker (p = 0.029) as source of communication, where more respondents in Dan Phuong stated these as important. Nevertheless, there was no significant difference between the two districts and stating internet and communication material as an important source. Nine households (6.98%) also mentioned other sources of communication about dengue fever, which included neighbours, other people in their village or through seminars.

### Knowledge about dengue fever symptoms and transmission

**Fig 3:**
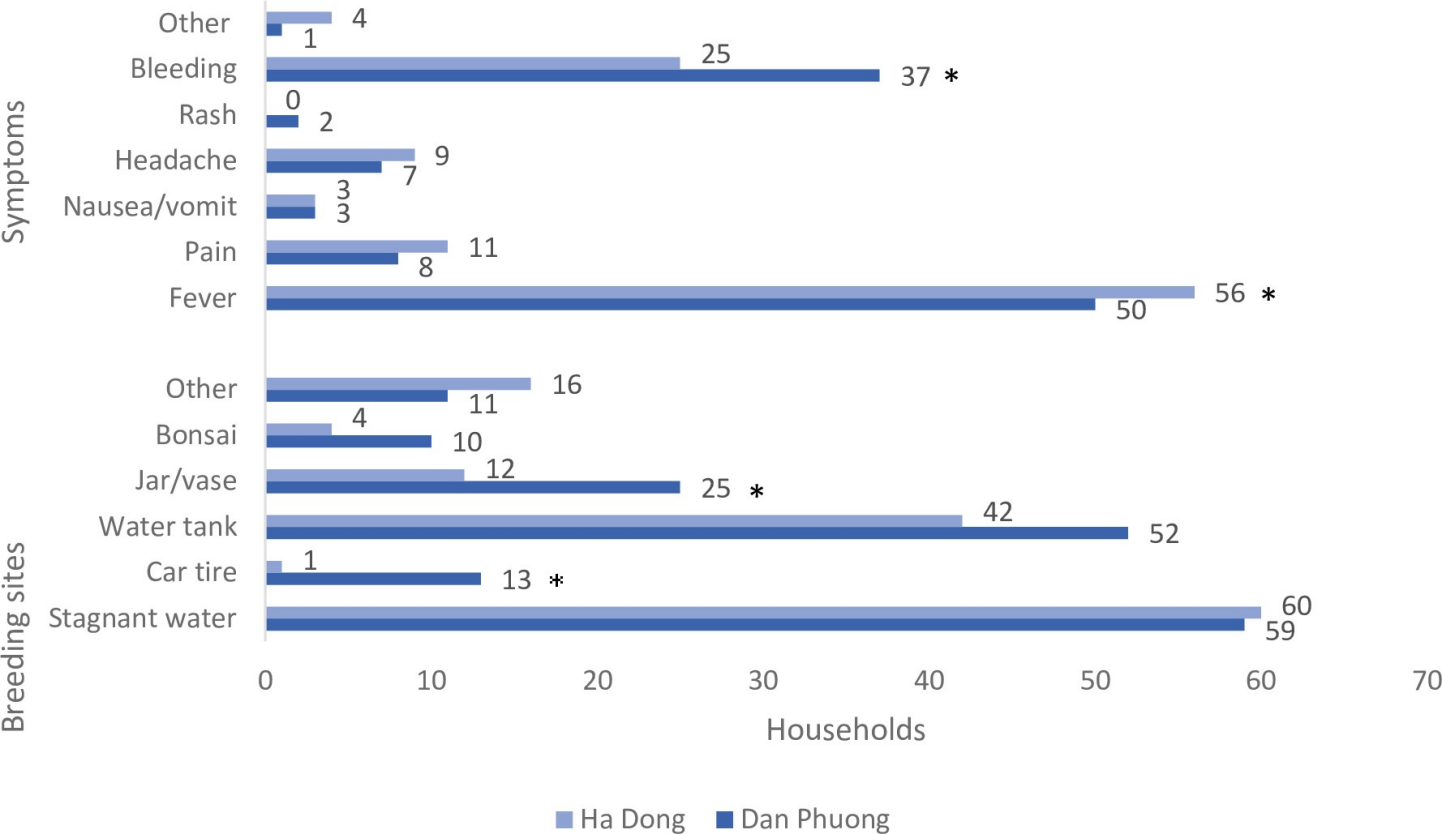
The number of households in each district that had knowledge about dengue fever symptoms and mosquito breeding sites. There were 71 households interviewed in Dan Phuong and 69 households in Ha Dong. *p<0.05 (Chi^2^ test).

Fever (96.4%) and bleeding (55.9%) were the most commonly mentioned symptoms of dengue virus infection in both districts. The other main symptoms, including pain (17.1%), nausea/vomiting (5.4%), headache (14.4%) and rash (1.8%) were less mentioned in both districts. There was a significant difference between the two districts in mentioning fever (p = 0.038) and bleeding (p = 0.048); respondents in Dan Phuong more often mentioned bleeding and respondents in Ha Dong more often mentioned fever (Fig 3).

Regarding the transmission of dengue virus, 126 of the households (90%) stated that they knew how dengue virus was transmitted and almost all of those (98.89%) stated the correct answer. However, one household (1.11%) located in Dan Phuong stated that dengue fever is transmitted through food. Out of the 14 households (10%) that did not know how dengue is transmitted, seven were located in Ha Dong and Dan Phuong, respectively.

### Knowledge about mosquito breeding sites

Most households (95.7%) reported knowledge of breeding sites for mosquitoes. Stagnant water (89.5%) and water tanks (70.7%) were the most commonly mentioned breeding sites in both districts. Bonsai (7.6%), jar/vase (27.8%) and car tires (10.5%) were less mentioned by all respondents. A significant difference was observed between the two districts and knowing if car tires (p = 0.001) and jar/vases (p = 0.019) can function as breeding sites for mosquitoes. 19.1% of the households in Dan Phuong and 1.5% of the households in Ha Dong mentioned car tires as a breeding site. 35.2% of the households in Dan Phuong and 17.4% of the households in Ha Dong mentioned jars or vases as important breeding sites. 20.3% of all households mentioned other mosquito breeding sites, e.g. sewer, contaminated water, the garden, garbage and bushes (Fig 3).

### Perceptions on dengue

**Fig 4:**
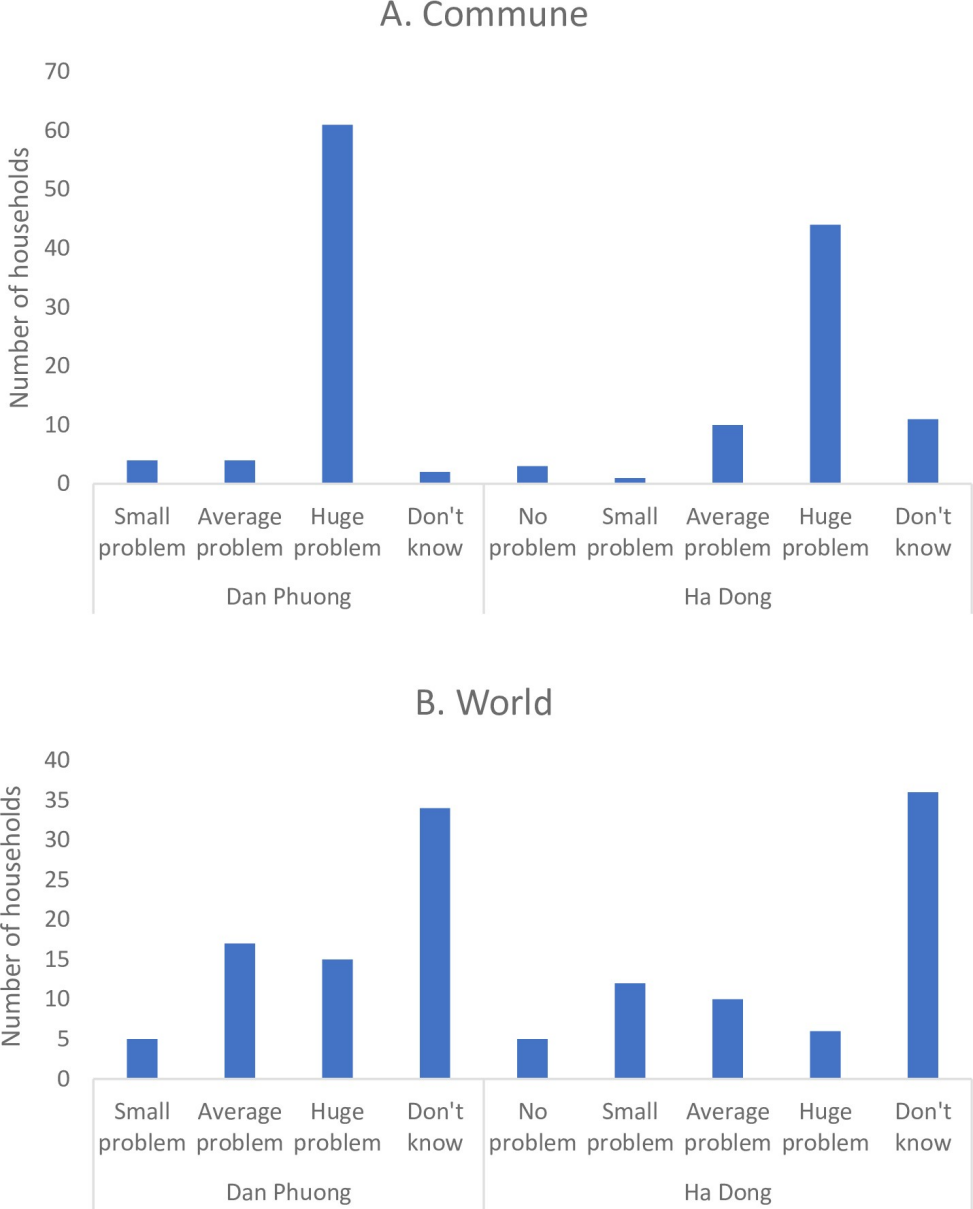
The dengue problem graded by the respondents in Dan Phuong and Ha Dong. A. The dengue problem graded in the respondent’s commune. B. The dengue problem graded in the world.

When the respondents were asked to grade the dengue situation in their commune, district, Hanoi, Vietnam and the world, a clear trend was observed for the two districts. The respondents living in Dan Phuong were grading dengue as an average or huge problem to a larger extent, as compared to the respondents living in Ha Dong. The respondents living in Ha Dong did generally grade dengue as a smaller problem or did not know if dengue fever is a problem. Another trend that was observed for the respondents in both districts was that more people generally had some estimation of whether dengue fever is a problem in their commune and/or district. However, when asked if dengue fever is a problem in the world, half of the respondents did not know (Fig 4).

### Dengue fever prevention practices

**Fig 5:**
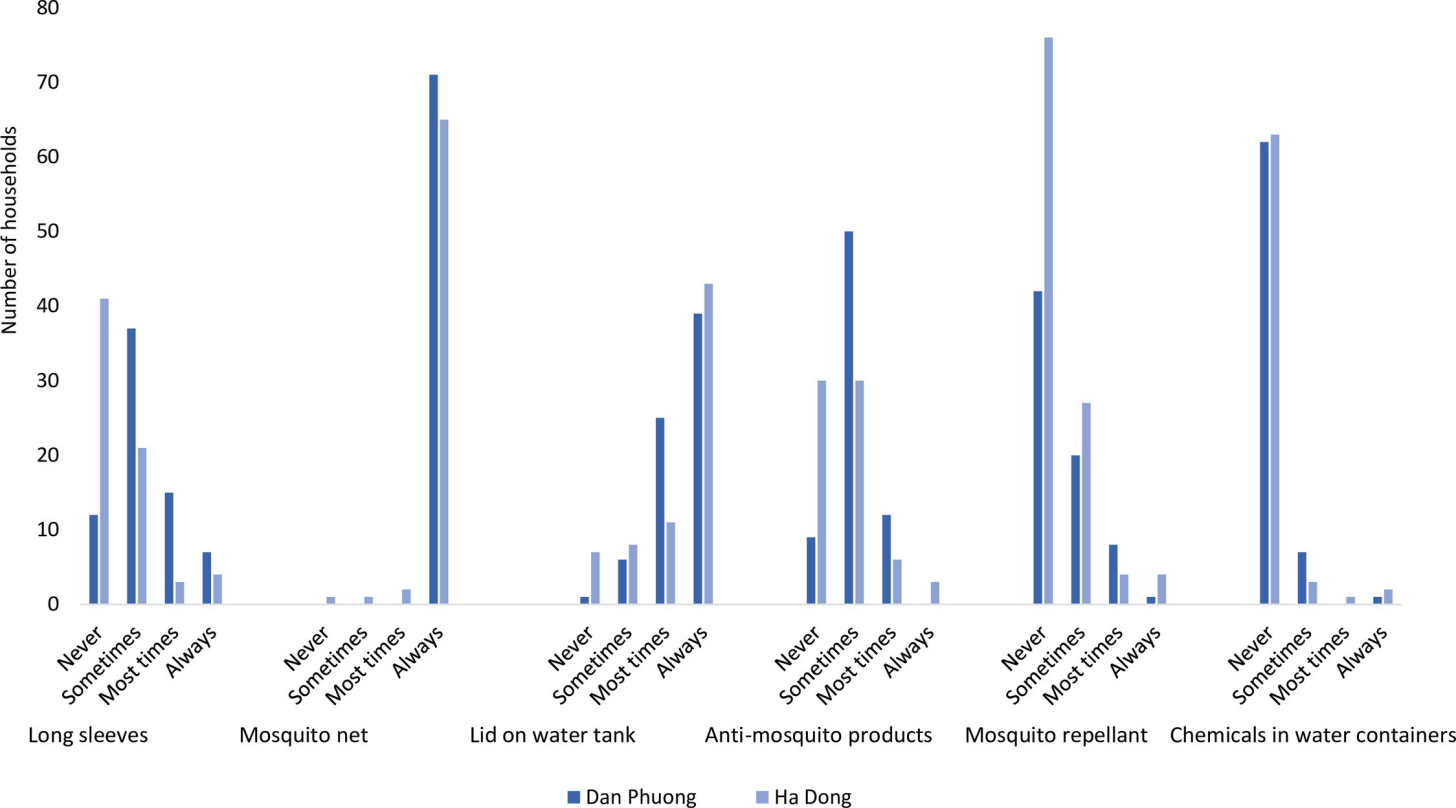
The number of households that protected themselves against mosquitoes in Ha Dong and Dan Phuong. *p<0.05 within the category between the districts, ***p<0.001 within the category between the districts.

Concerning dengue fever prevention practices, the respondents were asked about different methods of protection against mosquitoes. There was only a significant difference observed between the two districts regarding the usage of long sleeves (p<0.001), putting a lid on water tanks (p = 0.015) and the usage of anti-mosquito products (p<0.001), in which people living in Dan Phuong more often used these methods for mosquito protection. However, there was no significant difference between the two districts regarding using mosquito repellent, chemicals in the water and mosquito nets. Nevertheless, all households (100%) in Dan Phuong stated that they always use mosquito net, as compared to 94.2% of the households in Ha Dong (Fig 5).

### Mosquito and larvae collection

There were in total 3899 mosquitoes collected in the two districts, of which only 52 mosquitoes were of *Aedes* species, either *Ae*. *aegypti* or *Ae*. *albopictus* (Table 3). The *Aedes* mosquitoes were collected in three households in Dan Phuong and 11 households in Ha Dong. All *Aedes* mosquitoes collected in Dan Phuong were *Ae*. *albopictus* but only two of the *Aedes* mosquitoes in Ha Dong were *Ae*. *albopictus*. Out of the 3899 mosquitoes collected, 2684 (68.84%) mosquitoes were collected in livestock-keeping households and 1215 (31.2%) of the mosquitoes were collected in non-livestock keeping households.

**Table 3:** The number of mosquitoes and larvae collected in Dan Phuong and Ha Dong districts.

*District*	*Aedes spp mosquitoes (%)*	*Other mosquitoes (%)*	*Total mosquitoes (%)*	*Aedes spp larvae (%)*	*Culex spp larvae (%)*	*Total larvae (%)*
***Dan Phuong***	3* (5.77)	2530 (65.77)	2533 (64.97)	15 (28.85)	117 (50.21)	132 (46.32)
***Ha Dong***	49* (94.23)	1317 (34.23)	1366 (35.03)	37 (71.15)	116 (49.79)	153 (53.68)
***Total***	*52*	*3847*	*3899*	*52*	*233*	*285*

*2 of the *Aedes* spp mosquitoes in Ha Dong, and all 3 in Dan Phuong were *Aedes albopictus*

In the two districts, 285 larvae were collected, of which 52 of the larvae were of *Aedes* genus, and 37 out of these *Aedes* spp. larvae were collected in Ha Dong. There were 233 *Culex* spp. larvae and no *Anopheles* spp. larvae collected (Table 3).

A significantly larger proportion of the households in Ha Dong (16%) had *Aedes* spp. mosquitoes, as compared to 4% of the households in Dan Phuong (p = 0.02). In total, 14 of the households (10%) had *Aedes* spp. mosquitoes in the two districts. However, there was no significant association between livestock keeping and the presence of *Aedes* spp. mosquitoes in the households (p = 0.955) (Table 4). Similarly, no association could be detected when livestock keeping was split into pig keeping, poultry keeping or keeping of ruminants.

**Table 4:** The number of households with and without *Aedes* spp. mosquitoes in each district.

*Households with and without Aedes spp*. *mosquitoes*			
*District*	*No Aedes (%)*	*Aedes (%)*	P value*
*Dan Phuong*	67 (95.71)	3 (4.23)	0.02
*Ha Dong*	58 (84.06)	11 (15.94)	
***Livestock keeping***			
*No*	64 (90.14)	7 (9.86)	0.93
*Yes*	61 (89.71)	7 (10.14)	
***Total***	*126 (90)*	*14 (10)*	

*Chi^2^ test

### Dengue fever cases

**Fig 6:**
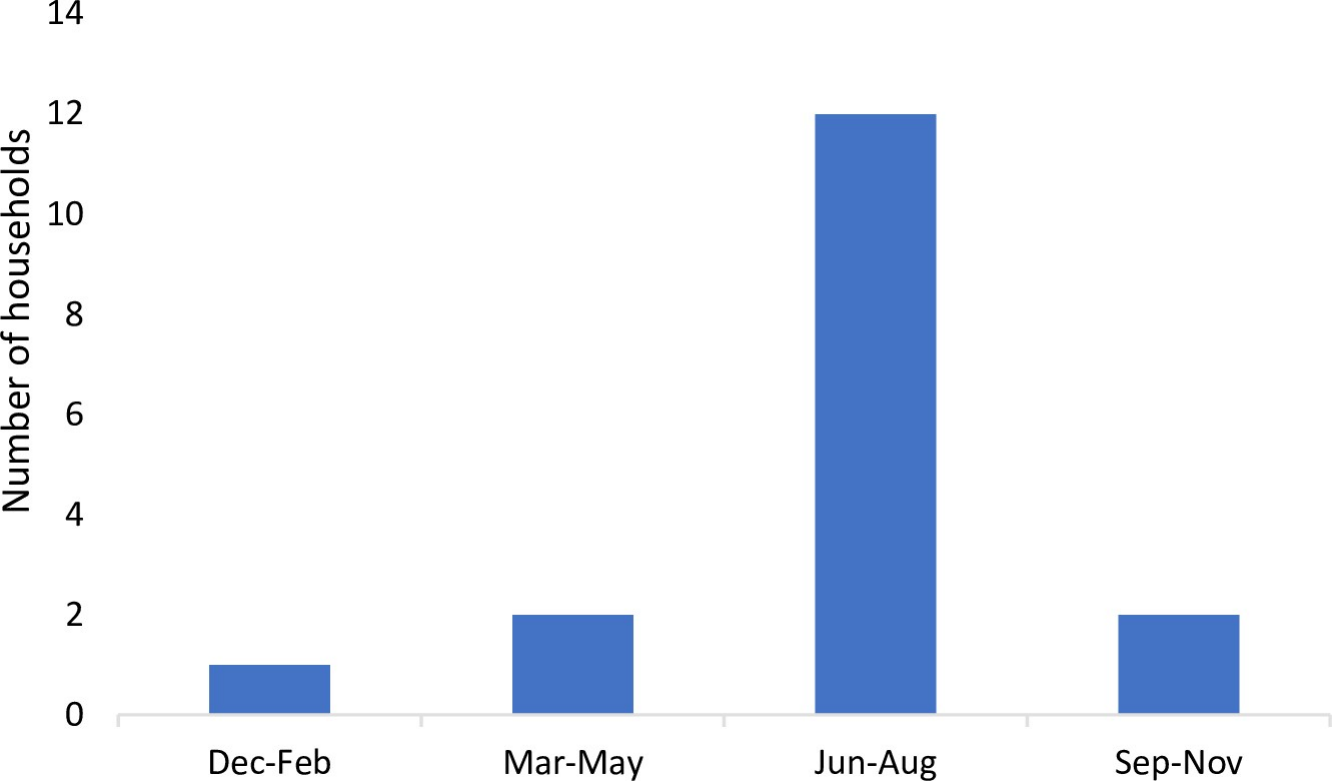
The number of households where at least one person had been diagnosed with dengue fever during different time periods of the year.

The respondents were asked if anyone in the household had been diagnosed with dengue fever during 2017 and there were significantly more households in Ha Dong (21.7%) where a member had been diagnosed with dengue fever, as compared to Dan Phuong (2.8%, p = 0.001). In total, 17 households (12.1%) had been diagnosed with dengue fever in 2017 (Table 5). Out of the 17 households, 12 of them (70.6%) had been diagnosed in the period of June to August 2017 (Fig 6). Within each household, the number of infected people varied. Twelve (16.9%) of the households that had been diagnosed with dengue fever during 2017 were non-livestock keeping, and 5 (7.25%) of the households were livestock-keeping. There were no significant association between the households that had been diagnosed with dengue during 2017 and the households keeping livestock (p = 0.08). There was however a significant association between the households having a case of dengue fever during 2017 and the number of *Aedes* spp. mosquitoes found in the households (p = 0.047), but no significant association with the number of *Aedes* spp. larvae (p = 0.523).

**Table 5:** The number of households with confirmed dengue fever cases in 2017 by district, livestock keeping, the presence of *Aedes* spp. mosquitoes and *Aedes* spp. larvae.

*Households with confirmed dengue fever in 2017*			
	*No (%)*	*Yes (%)*	P value*
***District***			
*Dan Phuong*	69 (97.18)	2 (2.82)	0.001
*Ha Dong*	54 (78.26)	15 (21.74)	
***Livestock keeping***			
*No*	59 (83.1)	12 (16.9)	0.08
*Yes*	64 (92.75)	5 (7.25)	
***Aedes spp*. *mosquitoes***			
*No*	113 (89.68)	13 (10.32)	0.047
*Yes*	10 (71.43)	4 (28.57)	
***Aedes spp*. *larvae***			
*No*	114 (88.37)	15 (11.63)	0.523
*Yes*	9 (81.82)	2 (18.18)	
***Total***	*123 (87*.*86)*	*17 (12*.*14)*	

*Chi^2^ test

The results of the multivariable analyses showed a higher risk to have dengue cases in the household if the household was in Ha Dong, or had better knowledge, while a reduced risk was found when the household kept livestock (Table 6).

**Table 6:** Results from the multivariable logistic model on risks of a household experiencing dengue fever cases.

	*Odds ratio*	*95% confidence interval*	P value*
***District***			
*Dan Phuong*	Reference		
*Ha Dong*	28.37	4.18–192.65	0.001
***Livestock keeping***			
*No*	Reference		
*Yes*	0.19	0.05–0.73	0.016
***Number of correctly identified symptoms or breeding grounds***			
*Per correct answer*	1.92	1.27–2.89	0.002
***Constant***	0.002	0.0001–0.322	<0.001

## Discussion

The increasing urbanization worldwide results in humans and animals living in close contact, which might increase the risk for infectious diseases, including vector-borne diseases. Livestock keeping may influence the number of vectors close to humans through creating new breeding sites for mosquitoes. This study found no association between livestock-keeping and dengue fever cases, or between livestock-keeping and the presence of the dengue mosquito vector *Aedes* spp.

When interviewing households in two districts of Hanoi, Dan Phuong and Ha Dong, a general trend was observed; people living in Dan Phuong had a better knowledge of dengue fever, attitude, and practice as compared to the people living in Ha Dong. It was also observed that health care workers (p = 0.03) and loudspeakers (p<0.001) were reported as significantly more important sources of communication for dengue fever in Dan Phuong, which could be one explanation for the greater awareness of dengue fever in this district. It would, therefore, be of importance to increase the awareness and put more effort into dengue fever prevention campaigns in Ha Dong and other central districts. In the multivariable analyses, higher knowledge was associated with higher risk for dengue fever cases in the household, but this analysis is not including causation, and therefore it can also be that having experiencing dengue fever in a household contributes to better knowledge. The respondents in Ha Dong, furthermore, reported more dengue fever cases as compared to Dan Phuong, which may be an effect of the respondents in Dan Phuong possessing better knowledge regarding dengue fever and therefore better mosquito control practices. It is possible that these results are biased by differences between the people interviewed in the two districts. However, our questions on demographics could not detect this, although there was a non-significant trend that people in the peri-urban Dan Phuong was slightly higher educated.

Another explanation for the higher number of dengue cases, could be the much higher population density in Ha Dong (6037.3 persons/km^2^), as compared to the lower population density of 1996.1 persons/km^2^ in Dan Phuong. Since humans are the main amplifying host of dengue virus, a higher population density will therefore facilitate the transmission of dengue virus and consequently the likelihood of getting infection is higher. The results from this study are in accordance with the general thought that people living in urban areas are more at risk for getting dengue fever [22]. However, another study previously performed in Vietnam by Schmidt et al. [23] concluded that people living in rural areas also has a high risk for getting infected with dengue virus due to more often using water storage containers and lacking access to tap water, and therefore, rural areas might contribute as much to the spread of dengue virus as urban areas. In this study, we found fewer *Aedes* spp in the peri-urban area, but also much better knowledge and more protective measures taken by the population. There can be a clustering effect of dengue, with more infections likely to occur around a primary case [24]. However, another study could not see this effect for clinical dengue, although people closer to a dengue case were more likely to have IgM towards the virus [20]. The same study also did not find a difference in infection rate in mosquitoes close to cases and controls.

English publications on the knowledge of dengue fever, attitude and practice studies performed in Vietnam, as well as studies about how livestock keeping in urban settings is affecting the presence of dengue vectors, are sparse. However, a household study performed in the Binh Thuan province (south of Vietnam) reported similar results as observed in this study; respondents living in low incidence areas had a better knowledge of dengue fever and better preventive practices. It was also found that TV was the most important source of information on dengue fever, similar to what was found in this study [15]. Our results also indicated that communication materials were deemed to be of little importance, which is consistent with a recent meta-analysis showing that interventions only distributing written communication materials had little effect on the knowledge and practices [25].

Dan Phuong is a peri-urban area with higher livestock density compared to the urban district Ha Dong. There was no association between the keeping of livestock and confirmed dengue fever cases in 2017 (p = 0.08), and there were also no association between the keeping of livestock and the presence of *Aedes* mosquitoes.

No association between livestock keeping and dengue virus infections or livestock-keeping and the presence of *Aedes* spp. mosquitoes could be demonstrated by this study, although the multivariable analyses indicated that households with livestock are less likely to experience dengue cases. Humans, animals and vectors share the environment and are closely interacting with each other. Their close relationship promotes the transmission of several infectious diseases. It could therefore still be an association between livestock keeping in urban environment and other mosquito-borne diseases, for example, JE. A study performed in southern Vietnam was investigating the occurrence of JE mosquito vectors in relation to the keeping of pigs in urban environments. More JE vectors were found in urban environments when farming pigs [17,26]. The results from that study indicated that there is an association between urban livestock keeping and the increased risk of mosquito-borne infections. Similarly, in the present study, the total number of mosquitoes was higher in livestock-keeping households.

Both adult mosquitoes and larvae were collected during the field visit. In general, there were less *Aedes* spp. mosquitoes collected as compared to other mosquito species in the both Dan Phuong and Ha Dong districts. For some households, no *Aedes* spp. mosquitoes were collected, but *Aedes* larvae were found. This may indicate that the method used for collection of mosquitoes was not optimal. Another important factor to be considered with the backpack aspirator as a collection method, is the time difference of the mosquito collection between the households. Different mosquito species are active during specific time points during the day, and *Aedes* spp. mosquitoes are especially active in the morning and/or twilight even though the mosquitoes also bite during day time [27,28]. During the fieldwork, the majority of the households were visited during day time, however, the time varied between the households. In a study by Ndenga et al. [29], significantly more *Aedes aegypti* mosquitoes had been collected in the afternoon as compared to the morning. Since the use of backpack aspirators allows targeting resting mosquitoes, this is however believed to have played a minor role for this study.

The low abundance of *Aedes* spp. is likely explained by the time of the year when the study was conducted. The field study was performed during March, a month with a low incidence of both mosquitoes and dengue virus infections in Hanoi. There is a seasonal pattern of dengue fever incidence in Vietnam and the peak of cases in Hanoi occurs between June-November, during the rainy period. Subsequently, there is a sudden decrease in the number of cases, followed by a slow increase in June [13,19,30]. The results from this study also confirms that the majority of the dengue virus infections occurs during the rainy season, since most of the cases in this study (70%) had been diagnosed during the period of June to August. However, this study clearly showed that *Aedes* vectors were present during the low season in both larval and adult stages indicating that breeding is on-going, and that risk is not negligent. This may contribute to our understanding of the vector ecology, which is still very much needed, since there are still doubts as to the links between vector collections and the risk for human cases [31]. Interestingly, this study could still find an association with households having experienced dengue fever being more likely to have *Aedes* mosquitoes, than other households, even though the cases occurred another time. However, the fact that the mosquito collection did not occur during the peak season for dengue fever still affected the results of the comparisons between the areas, and analyses on species level could not be done due to the low number of mosquitoes. More large-scale studies over different seasons are therefore warranted.

Vietnam is highly affected by dengue fever and the cornerstone for dengue fever control is to control the mosquito vector since treatment and other prevention options are lacking. There is therefore an increasing need for new methods to control dengue fever. There has been some major initiatives on dengue control in Vietnam to manage this problem. One example is the usage of *Mesocyclops* (copepod) in large water containers to reduce the *Aedes* larvae populations that has been used in community programs in several provinces of Vietnam. In a study by Nam et al. [32], *Ae*. *aegypti* larvae was reduced by approximately 90% after one year and eliminated in two out of three communes at the end of the study, three years from the start. Similarly, the incidence of dengue fever was also reduced in the communes that participated in the study despite considerable number of dengue fever cases were reported in surrounding districts [32–34].

In conclusion, people living in the peri-urban Dan Phuong district, keeping more livestock, had a better knowledge on dengue fever, attitude and practice as compared to the people living in the urban Ha Dong district. There was an association between living in the more urban Ha Dong and the presence of *Aedes* spp. mosquitoes and the risk for getting infected with dengue virus. One of the most important findings here is the difference in protection against mosquitoes in the two areas, and also the importance of different media, particularly TV, as a mean of dissemination of information. In this study, no association between the presence of livestock and *Aedes* spp. mosquitoes/larvae could be observed during the season of lowest occurrence of dengue. However, there is still a need for further investigations regarding the association between urban livestock management and mosquito-borne infections and the possible risks associated with it.

## Supporting information

S1 Text Strobe checklist. (DOC)

S2 Text Questionnaire in English. (DOCX)

S3 Text Questionnaire in Vietnamese. (DOCX)

S1 Data Data set for evaluation of knowledge, attitudes and practices. (XLS)
