# Supplementary material for: Urban livestock-keeping and dengue in urban and peri-urban Hanoi, Vietnam
Source: PLoS Negl Trop Dis. 2019 Nov 26;13(11):e0007774. doi: 10.1371/journal.pntd.0007774 (PMC6879131; doi:10.1371/journal.pntd.0007774)
Supplement: S2 Text — (DOCX) [file pntd.0007774.s002.docx]

**
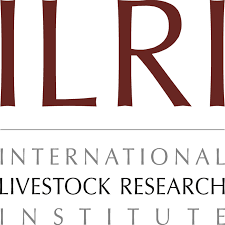
**
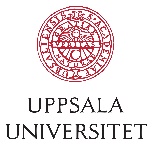
**Dengue awareness study**

**INTRODUCTION**

Good morning / Good afternoon. My name is _________________ and I work for the International Livestock Research Institute (ILRI) which is based in Hanoi. We are conducting a study on how the presence of mosquitoes is affected by the keeping of the livestock in urban environments. This study has two parts 1) baseline survey to help understand the awareness of dengue and 2) to collect mosquitoes and investigate if the mosquitoes are infected with Dengue virus. We are visiting you because your household has been selected randomly to participate in the dengue awareness study. If you accept to participate in the dengue awareness study, we will ask you a few questions related to your household, heard and awareness about dengue. We will also request you to get the consent to place mosquito traps on your land for further laboratory analysis.

**Potential risks**

Your participation in this evaluation has no physical risk. We maintain strict control of all the information gained from staffs.

**Potential benefits**

This is an important study to control the burden of neglected tropical diseases such as dengue fever in the community.

**Confidentiality**

The research team promises to respect privacy and confidentiality of your information. This information we talk about will be shared with our research team members, but we will remove all names so that no one be able to trace back the information to you. If you voluntarily participate in this study, you may withdraw at any time without consequences of any kind. You may use the option of removing your data from the study. You may also refuse to answer any questions you don’t want to answer and still remain in the study. The investigator may withdraw you from this research if circumstances arise that warrant doing so. You are not waiving any legal claims, rights or remedies because of your participation in this research study.

If you have any questions now or later you are welcome to call the researchers:

- **Dr Johanna Lindahl** 0718-929937
- **Mr. Nguyen Tien Thang** 0981582712

If you have questions regarding your rights as a research participant, contact:

**Institutional Review Board**, Room 103 Building A – Hanoi University of Public Health
No 1A, Duc Thang str, Duc Thang ward, Bac Tu Liem district, HaNoi
Tel: 024 6266 3024. Email: irb@huph.edu.vn

Questions to households

**Household id: ________**

**1. General questions**

| 1. **Respondent details**: | Relationship with household head: | Gender: | Age: |
| --- | --- | --- | --- |
|  | Highest level of education:  [ ]  1 = no education  2 = primary school  3 = secondary school  4 = high school  5 = college/university | Occupation:  [ ]  Define 3: _________________  1 = farmer  2 = any medical profession/medical education 3 = Others | |
| 1. **If respondent is not household head:** | Gender of household head: | Age of household head: | |
| 1. **Household details:** | Number of people living* in the household:  [ ] | Number of children living* in the household: [ ] | |
|  | Number of children under 15 years of age living* in the household:  [ ] |  | |

* people living in the household is defined as living in the household continuously for at least 2 months

**2. Herd details**

**2.1** Do your household own livestock?
 At least one larger livestock [ ] At least 10 smaller livestock [ ] No livestock [ ]
 (If no livestock is marked, skip part 2.2)

**2.2** Details

| **Animal:** | **Amount:** | **Animal husbandry:  1 = indoor, 2 = tied up, partly outdoor 3 = fenced in outdoor 4 = free roaming** | **Any disease in the last year? If yes, describe symptoms.** |
| --- | --- | --- | --- |
| **Pig:** |  |  |  |
| **Chicken:** |  |  |  |
| **Buffalo:** |  |  |  |
| **Duck:** |  |  |  |
| **Goat:** |  |  |  |
| **Cattle:** |  |  |  |
| **Other:** |  |  |  |

**3. Awareness about Dengue fever**

**3.1.1** Have you heard about diseases being transmitted from mosquitoes to humans? ________ [Yes/No]

|  |
| --- |

**3.1.2** If yes, can you name the disease(s) you think can be transmitted from mosquitoes?
 Malaria [ ] Zika [ ] Dengue fever [ ] Japanese encephalitis [ ] Others [ ]
 If others were marked, please define other diseases mentioned:

**3.2.1** Have you heard about Dengue fever? ________ [Yes/No]

**3.2.2.1** If yes, what have you heard of the following?
 Definition [ ] Symptoms [ ] Preventive measures [ ] Treatment [ ]

**3.2.2.2** If yes, what are the sources of information you have heard about dengue of the following?
 TV [ ] Health workers [ ] Communication materials [ ] Internet [ ]
 Loud speaker [ ] Others [ ]
 If others were marked, please define where:

|  |
| --- |

**3.3.1** Do you know the main symptoms of Dengue fever? __________ [Yes/No]

**3.3.2** If yes, which symptoms have you heard of?
 High fever [ ] Muscle pains [ ] Nausea/vomiting [ ] Severe headache [ ] Rash [ ]
 Bleeding [ ] Other [ ]

If others were marked, please define other symptoms:

|  |
| --- |

**3.4.1** Do you know how Dengue fever is transmitted to humans? __________ [Yes/No]

**3.4.2** If yes, how?

|  |
| --- |

**3.5.1** Do you know any breeding sites for mosquitoes? ________ [Yes/No]

**3.5.2** If yes, what breeding sites do you know?
 Stagnant water containers [ ] Car tires [ ] Water tanks [ ] Jar/vase [ ] Bonsai [ ]
 Others [ ]
 If others were marked, please define others:

|  |
| --- |

**3.6** Can you mention some ways to prevent yourself from getting Dengue fever?

|  |
| --- |

**3.7** Which of the following methods of protection do you use?

| **Method:** | **How often? 1 = Never,**  **2 = Some time, 3 = Most time,**  **4 = Always** | **Define the product/chemical used:** |
| --- | --- | --- |
| **Long sleeves** |  |  |
| **Mosquito net** |  |  |
| **Mosquito repellent** |  |  |
| **Keep lids on water tanks** |  |  |
| **Chemical in water containers** |  |  |
| **Anti-mosquito products (e.g. insecticides)** |  |  |
| **Others:** |  |  |

**3.8.1** Do you know any risk factors for getting Dengue fever? ________ [Yes/No]

**3.8.2** If yes, which risk factors do you think is important of the following?
 Warm and humid season [ ] High population density [ ] Stagnant water containers [ ]
 Others [ ]

|  |
| --- |

If others were marked, please define other risk factors you have heard of:

**Note: Interviewee can only suggest about the weather and population density.**

**3.9.1** Have anyone in the family had any of the following symptoms:

| **Symptoms:** | **During the last 2 weeks [Yes/No]:** | **During the last 6 months [Yes/No]:** | **How was it treated?**  **1 = not treated,**  **2 = self-treated,**  **3 = medical help** | **Were the symptoms diagnosed?**  **[Yes/No]** | **If diagnosed, by who?**  **1 = private doctor,**  **2 = medical care**  **3 = family member** |
| --- | --- | --- | --- | --- | --- |
| **High fever** |  |  |  |  |  |
| **Severe headache** |  |  |  |  |  |
| **Muscle and joint pains** |  |  |  |  |  |
| **Nausea** |  |  |  |  |  |
| **Vomiting** |  |  |  |  |  |
| **Swollen glands** |  |  |  |  |  |
| **Rash** |  |  |  |  |  |

**3.9.2** If any of the symptoms were diagnosed in 3.9.1, which diagnose? _________________________

**3.10.1** Has someone in the family been diagnosed with Dengue fever in 2017? ________ [Yes/No]

**3.10.2** If yes, during what month? _____________________________________________

**3.11.1** Do you know anyone/heard of anyone (beside family members) that has been infected with
 Dengue? ________ [Yes/No]

**3.11.2** If yes, who? ______________________________________________________
 **3.12.1** How big is the problem with Dengue fever?
 In your commune [ ] In your district [ ] In Ha Noi [ ] In Viet Nam [ ] In the world [ ]

*1 = Not a problem, 2 = Small problem, 3 = Average problem, 4 = Huge problem, 5 = I don’t know*

**4. Checklist**

- 1. Are there any water-filled open containers outside the house, on the premises?

Yes [ ] How many________ No [ ] Not applicable/no outside area [ ]

- 1. Can any larvae be observed in these?

Yes [ ] In how many________ No [ ] Larvae collected [ ]

- 1. Are there any water-filled open containers inside the house, on the premises?

Yes [ ] How many________ No [ ]

- 1. Can any larvae be observed in these?

Yes [ ] In how many________ No [ ] Larvae collected [ ]

**Informed consent form**

**INFORMED CONSENT FORM**

~not to be attached in the questionnaire~

Do you have any questions about the research we wish to conduct? Once again, we thank you for accepting us in your household and now wish to ask for your availability to participate in the study. Please note that your participation in the study is voluntary and that you can withdraw your participation at any time if you wish. We assure you that whatever information you share with the research team is confidential.

Are you willing to be part of this study?

| We respect your choice and do appreciate your participation | | | |
| --- | --- | --- | --- |
|  | | Respondent’s Initials | signature |
| YES | Verbal |  |  |
|  | Written |  |  |
| NO |  |  |  |
|  |  |  |  |
