# Supplementary material for: Urban livestock-keeping and dengue in urban and peri-urban Hanoi, Vietnam
Source: PLoS Negl Trop Dis. 2019 Nov 26;13(11):e0007774. doi: 10.1371/journal.pntd.0007774 (PMC6879131; doi:10.1371/journal.pntd.0007774)
Supplement: S3 Text — (DOCX) [file pntd.0007774.s003.docx]

**
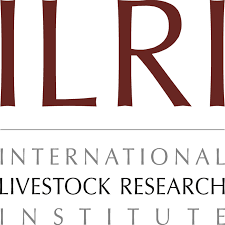

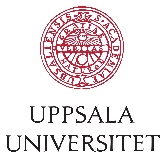
**

**Nghiên cứu đánh giá nhận thức về bệnh sốt Dengue**

**GIẤY ĐỒNG Ý THAM GIA NGHIÊN CỨU**

**GIỚI THIỆU NGHIÊN CỨU**

Xin chào ông bà. Tên tôi là _________________. Tôi đang làm việc tại Viện nghiên cứu Chăn nuôi Quốc tế (ILRI) ở Hà Nội. Chúng tôi đang tiến hành nghiên cứu về ảnh hưởng của chăn nuôi gia súc gia cầm tới quần thể muỗi ở khu vực đô thị. Nghiên cứu này sẽ gồm 2 phần: 1) tìm hiểu nhận thức của người dân về bệnh sốt dengue và 2) thu thập mẫu muỗi trưởng thành và thí nghiệm muỗi có virus Dengue hay không. Gia đình ông bà được chọn để tham gia nghiên cứu đánh giá nhận thức về bệnh sốt Dengue. Nếu ông bà đồng ý tham gia vào nghiên cứu này, chúng tôi sẽ hỏi ông bà một vài câu hỏi về nhận thức và hành vi của ông bà liên quan đến bệnh sốt xuất huyết. Chúng tôi cũng sẽ đề nghị được đặt bẫy muỗi trong và ngoài nhà của ông bà để lấy mẫu và phân tích trong phòng thí nghiệm.

**Các nguy cơ tiềm ẩn**

Sự tham gia của ông bà trong nghiên cứu này không có nguy cơ nào. Nhóm nghiên cứu sẽ kiểm soát chặt chẽ quá trình tiến hành nghiên cứu.

**Các lợi ích tiềm năng**

Đây là một nghiên cứu quan trọng để góp phần kiểm soát gánh nặng các bệnh lây nhiễm như sốt xuất huyết ở cộng đồng.

**Tính bảo mật**

Nhóm nghiên cứu sẽ đảm bảo về quyền riêng tư và bảo mật các thông tin của ông bà. Các thông tin trao đổi khi phỏng vấn sẽ chỉ được chia sẻ ở trong nhóm nghiên cứu nhưng chúng tôi sẽ xóa tên của ông bà để không ai có thể biết được thông tin của ông bà. Nếu ông bà tình nguyện tham gia vào nghiên cứu, ông bà có thể rời khỏi nghiên cứu bất cứu thời điểm nào mà không bị ảnh hưởng gì. Ông bà cũng có thể chọn xóa thông tin của mình từ nghiên cứu. Ông bà cũng có quyền từ chối trả lời bất kì câu hỏi nào mà mình không muốn trả lời và vẫn tiếp tục tham gia nghiên cứu. Điều tra viên có thể rút tên ông bà khỏi nghiên cứu trong trường hợp cần thiết. Ông bà không phải từ bỏ bất kì yêu cầu pháp lý hay quyền khi tham gia nghiên cứu này.

Nếu ông bà có bất kì câu hỏi nào liên quan đến nghiên cứu này, xin hãy liên lạc với chúng tôi :

TS. Johanna Lindahl 0718-929937

Nguyễn Tiến Thắng 0981582712

Nếu ông bà có bất kì câu hỏi nào về quyền của mình khi tham gia nghiên cứu, xin liên hệ:

## **Hội Đồng Đạo đức Đại học Y tế Công cộng**, Phòng 103 Nhà A, Số 1A, đường Đức Thắng, phường Đức Thắng, Quận Bắc Từ Liêm, Hà Nội ĐT: 024 6266 3024. Email: irb@huph.edu.vn

Ông bà có bất kỳ câu hỏi nào về nghiên cứu này không? Một lần nữa, chúng tôi muốn cảm ơn ông bà và muốn hỏi ông bà có đồng ý để tham gia vào nghiên cứu của chúng tôi không? Sự tham gia của ông bà trong nghiên cứu là hoàn toàn tự nguyện và ông bà có thể rút khỏi nghiên cứu vào bất kì thời điểm nào ông bà muốn. Chúng tôi đảm bảo rằng các thông tin ông bà chia sẻ với nhóm nghiên cứu sẽ được bảo mật hoàn toàn.

Ông bà có sẵn lòng tham gia vào nghiên cứu này không?

| Chúng tôi tôn trọng sự lựa chọn của ông bà và cảm ơn sự hợp tác của ông bà | | | |
| --- | --- | --- | --- |
|  | | Ý kiến của người trả lời | Chữ kí |
| CÓ | Lời nói |  |  |
|  | Chữ viết |  |  |
| KHÔNG |  |  |  |
|  |  |  |  |

BỘ CÂU HỎI

Mã số hộ gia đình __________

**1. Câu hỏi chung**

| 1. **Thông tin người được phỏng vấn** | Quan hệ với chủ hộ: | Giới: | Tuổi: |
| --- | --- | --- | --- |
|  | Trình độ học vấn cao nhất:  [ ]  1 = không đi học  2 = học hết tiểu học  3 = học hết THCS  4 = học hết THPT  5 = học hết cao đẳng/đại học | Nghề nghiệp:  [ ]  Khác 3: _________________  1 = nông dân  2 = có bất kì chuyên môn về y tế  3 = Khác | |
| 1. **Nếu người trả lời không phải chủ hộ:** | Giới tính của chủ hộ | Tuổi của chủ hộ: | |
| 1. **Đặc điểm hộ gia đình:** | Số người sống trong hộ gia đình:  [ ] | Số trẻ em sống trong hộ gia đình: [ ] | |
|  | Số trẻ em dưới 15 tuổi:  [ ] |  | |

* Số người sống trong hộ gia đình được định nghĩa là sống ở hộ gia đình liên tục trong vòng 2 tháng

**2. Số lượng gia súc gia cầm trong nhà**

**2.1** Nhà ông bà có nuôi gia súc/gia cầm không?
 Ít nhất 1 loại gia súc [ ] Ít nhất 10 con gia cầm/vật nuôi nhỏ [ ] Không nuôi [ ]
 (Nếu không nuôi gia súc/gia cầm, bỏ qua phần 2.2)

| **Gia súc/gia cầm:** | **Số lượng:** | **Chăn nuôi:**  **1 = trong nhà, 2 = liền nhà, một phần ngoài trời, 3 = Có chuồng ở ngoài nhà, 4 = chăn thả tự do** | **Trong vòng một năm trở lại đây đàn gia súc/gia cầm có bị bệnh gì không? Miêu tả các triệu chứng nếu có.** |
| --- | --- | --- | --- |
| **Lợn:** |  |  |  |
| **Gà:** |  |  |  |
| **Vịt:** |  |  |  |
| **Trâu:** |  |  |  |
| **Bò:** |  |  |  |
| **Dê:** |  |  |  |
| **Khác:** |  |  |  |

**2.2** Chi tiết

**3. Nhận thức về bệnh lây truyền qua muỗi đốt và sốt xuất huyết**

**3.1.1** Ông bà đã bao giờ nghe về các bệnh truyền từ muỗi sang người chưa? ________ [Có/Không]

|  |
| --- |

**3.1.2** Nếu có, ông bà có thể kể tên bệnh nào truyền từ muỗi?
 Sốt rét [ ] Zika [ ] Sốt xuất huyết [ ] Viêm não nhật bản [ ] Khác [ ]
 Nếu chọn khác, xin hãy nêu rõ:

**3.2.1** Ông bà đã bao giờ nghe về bệnh sốt xuất huyết? ________ [Có/Không]

**3.2.2.1** Nếu có, ông bà nghe về các vấn đề gì sau đây? **(Nhiều lựa chọn và không đọc đáp án)**
 Định nghĩa [ ] Các triệu chứng [ ] Các cách dự phòng [ ]

Cách điều trị [ ] Cách lây truyền [ ]

**3.2.2.2** Nếu có, ông bà nghe từ những nguồn nào sau đây?
 TV [ ] Nhân viên y tế [ ] Các tài liệu truyền thông [ ] Internet [ ]
 Loa phát thanh [ ] Khác [ ]

Nếu chọn khác, xin hãy nêu rõ:

|  |
| --- |

**3.3.1** Ông bà có biết triệu chứng chính của sốt xuất huyết không? __________ [Có/Không]

**3.3.2** Nếu có, ông bà có thể kể tên triệu chứng nào? **(Nhiều lựa chọn và không đọc đáp án)**
 Sốt cao [ ] Đau cơ [ ] Buồn nôn/ói [ ] Đau đầu trầm trọng [ ] Ngứa [ ]
 Xuất huyết [ ] Khác [ ]

Nếu chọn khác, xin hãy nêu rõ:

|  |
| --- |

**3.4.1** Ông bà có biết sốt xuất huyết được lây truyền đến người như thế nào không? __________ [Có/Không]

**3.4.2** Nếu có, như thế nào?

|  |
| --- |

**3.5.1** Ông bà có biết những nơi sinh sản của muỗi không? ________ [Có/Không]

**3.5.2** Nếu có, kể tên những nơi sinh sản của muỗi mà ông bà biết? **(Nhiều lựa chọn và không đọc đáp án)**
 Thùng/xô/chậu/ vại chứa nước đọng [ ] Lốp xe [ ] Bể/chum nước [ ]

Lọ/Bình hoa [ ] Hòn non bộ/cây Bonsai [ ] Khác [ ]

Nếu chọn khác, xin hãy nêu rõ:

|  |
| --- |

**3.6.** Ông bà có thể kể một vài cách để phòng chống bệnh sốt xuất huyết?

|  |
| --- |

**3.7** Những biện pháp nào mà ông bà sử dụng để phòng chống sốt xuất huyết? **(Đọc từng đáp án)**

| **Biện pháp:** | **Mức độ thường xuyên?  1 = Không bao giờ,**  **2 = Thỉnh thoảng, 3 = Hầu như lúc nào cũng dùng,**  **4 = Luôn luôn** | **Tên sản phẩm/hóa chất sử dụng:** |
| --- | --- | --- |
| **Mặc áo dài tay** |  |  |
| **Dùng màn** |  |  |
| **Thuốc đuổi muỗi** |  |  |
| **Dùng nắp đậy các dụng cụ chứa nước** |  |  |
| **Dùng hóa chất trong dụng cụ chứa nước** |  |  |
| **Các sản phẩm diệt muỗi** |  |  |
| **Khác:** |  |  |

**3.8.1** Ông bà có biết những yếu tố nguy cơ nào có thể dẫn đến mắc sốt xuất huyết không? ________ [Có/Không]

|  |
| --- |

**3.8.2** Nếu có, kể tên những yếu tố nguy cơ mà ông bà biết?
 Thời tiết ấm và ẩm [ ] Mật độ dân cư cao [ ] Các dụng cụ chứa nước tù đọng [ ]
 Khác [ ]

Nếu chọn khác, xin hãy nêu rõ yếu tố nguy cơ khác mà ông bà được nghe:

**Lưu ý: Điều tra viên chỉ gợi ý và không đọc đáp án về thời tiết và mật độ dân số nếu người được phỏng vấn không đề cập đến**

**3.9.1** Đã có ai trong gia đình ông bà có những triệu chứng sau đây chưa: **(Đọc từng đáp án)**

| **Các triệu chứng:** | **Trong vòng 2 tuần trước [Có/Không]:** | | **Trong vòng 6 tháng trước [Có/Không]:** | | **Triệu chứng được chữa trị như thế nào?**  **1 = không chữa trị,**  **2 = tự điều trị,**  **3 = có sự điều trị từ bên y tế** | | **Các triệu chứng có được chẩn đoán không?**  **[Có/Không]** | | **Ai làm công việc chẩn đoán, nếu có?**  **1 = bác sỹ tư,**  **2 = cơ sở y tế công**  **3 = thành viên trong gia đình** | |
| --- | --- | --- | --- | --- | --- | --- | --- | --- | --- | --- |
| **Sốt cao** |  |  |  |  |  |  |  |  |  |  |
| **Đau đầu nặng** |  |  |  |  |  |  |  |  |  |  |
| **Đau cơ và khớp** |  |  |  |  |  |  |  |  |  |  |
| **Buồn nôn** |  |  |  |  |  |  |  |  |  |  |
| **Ói mửa** |  |  |  |  |  |  |  |  |  |  |
| **Viêm tuyến, sưng tấy** |  |  |  |  |  |  |  |  |  |  |
| **Ngứa** |  |  |  |  |  |  |  |  |  |  |

**3.9.2** Nếu có bất kỳ triệu chứng nào được nêu ở 3.9.1, bệnh được chẩn đoán là gì?

_________________________

**3.10.1** Có ai trong gia đình ông bà chẩn đoán mắc sốt xuất huyết năm 2017 không? ________ [Có/Không]

**3.10.2** Nếu có, trong khoảng thời gian tháng mấy? _____________________________________________

**3.11.1** Ông bà có biết ai hoặc đã từng nghe đến ai (ngoài thành viên trong gia đình) bị bệnh sốt xuất huyết chưa? ________ [Có/Không]

**3.11.2** Nếu có, ai bị mắc? ______________________________________________________

**3.12.1** Theo ông bà, bệnh sốt xuất huyết nghiêm trọng ở mức độ nào?

Ở xã/phường? [ ] Ở quận huyện? [ ] Ở Hà Nội nói chung? [ ] Ở Việt Nam? [ ] Trên thế giới? [ ]

*1 = Không phải là vấn đề, 2 = Vấn đề nhỏ, 3 = Trung bình, 4 = Vấn đề rất lớn , 5 = Tôi không biết*

## Danh mục

- 1. Có thể quan sát thấy số thùng/vại/chum đựng nước để ở ngoài nhà không?

Có [ ] Số lượng________ Không [ ] Không có khu vực sân vườn bên ngoài nhà [ ]

- 1. Có thể nhìn thấy loăng quăng bọ gậy trong thùng/vại/chum nước ngoài nhà không?

Có [ ] Số lượng________ Không [ ] Loăng quăng bọ gậy thu thập được [ ]

- 1. Có thể quan sát thấy số thùng/vại/chum đựng nước để ở trong nhà không?

Có [ ] Số lượng________ Không [ ]

- 1. Có thể nhìn thấy loăng quăng bọ gậy trong thùng/vại/chum nước trong nhà không?

Có [ ] Số lượng________ Không [ ] Loăng quăng bọ gậy thu thập được [ ]

Chúng tôi chân thành cảm ơn sự giúp đỡ của ông bà!
